# 5-Methyl-3-phenyl­isoxazole-4-carb­oxy­lic acid

**DOI:** 10.1107/S1600536813011410

**Published:** 2013-05-15

**Authors:** Chandra, N. Srikantamurthy, G. J. Vishalakshi, S. Jeyaseelan, K. B. Umesha, M. Mahendra

**Affiliations:** a Department of Studies in Physics, Manasagangotri, University of Mysore, Mysore 570 006, India; b Department of Chemistry, Yuvaraja’s College, University of Mysore, Mysore 570 005, India; c Department of Physics, St Philomena’s College, Mysore, India

## Abstract

In the title compound, C_11_H_9_NO_3_, the phenyl and isoxazole rings form a dihedral angle of 56.64 (8)°. The carb­oxy group is almost in the same plane as the isoxazole ring with a C—C—C—O torsion angle of −3.3 (2)°. In the crystal, pairs of O—H⋯O hydrogen bonds link the mol­ecules into head-to-head dimers. C—H⋯N hydrogen bonds and π–π stacking inter­actions between phenyl rings [centroid–centroid distance = 3.9614 (17)Å] link the dimers into a three-dimensional network.

## Related literature

For the biological and pharmaceutical importance of isoxazoles, see: Basappa *et al.*, (2003 ▶); Conti *et al.* (1998 ▶); Kang *et al.* (2000 ▶); Lee *et al.* (2009 ▶); Shin *et al.* (2005 ▶); Stevens & Albizati (1984 ▶). For bond-length and angle data in related structures, see: Wolf *et al.* (1995 ▶); Chandra *et al.*, (2013 ▶).
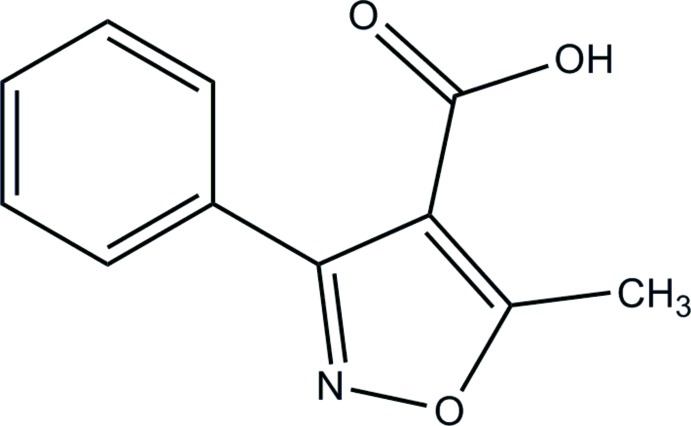


## Experimental

### 

#### Crystal data

C_11_H_9_NO_3_
*M* *_r_* = 203.19
Monoclinic, 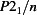

*a* = 11.953 (4) Å
*b* = 5.981 (2) Å
*c* = 14.142 (5) Å
β = 105.548 (6)°
*V* = 974.0 (6) Å^3^
*Z* = 4
Mo *K*α radiation
μ = 0.10 mm^−1^
*T* = 273 K
0.30 × 0.25 × 0.20 mm

#### Data collection

Bruker APEXII CCD area-detector diffractometer
8619 measured reflections
1712 independent reflections
1558 reflections with *I* > 2σ(*I*)
*R*_int_ = 0.026

#### Refinement

*R*[*F* ^2^ > 2σ(*F* ^2^)] = 0.039
*wR*(*F* ^2^) = 0.111
*S* = 1.05
1712 reflections
138 parameters
H-atom parameters constrained
Δρ_max_ = 0.19 e Å^−3^
Δρ_min_ = −0.14 e Å^−3^

### 

Data collection: *APEX2* (Bruker, 2009 ▶); cell refinement: *SAINT* (Bruker, 2009 ▶); data reduction: *SAINT*; program(s) used to solve structure: *SHELXS97* (Sheldrick, 2008 ▶); program(s) used to refine structure: *SHELXL97* (Sheldrick, 2008 ▶); molecular graphics: *PLATON* (Spek, 2009 ▶); software used to prepare material for publication: *SHELXL97*.

## Supplementary Material

Crystal structure: contains datablock(s) global, I. DOI: 10.1107/S1600536813011410/bg2504sup1.cif

Structure factors: contains datablock(s) I. DOI: 10.1107/S1600536813011410/bg2504Isup2.hkl

Supplementary material file. DOI: 10.1107/S1600536813011410/bg2504Isup3.cml

Additional supplementary materials:  crystallographic information; 3D view; checkCIF report

## Figures and Tables

**Table 1:** Hydrogen-bond geometry (Å, °)

*D*—H⋯*A*	*D*—H	H⋯*A*	*D*⋯*A*	*D*—H⋯*A*
O14—H14⋯O15^i^	0.82	1.81	2.6252 (18)	172
C11—H11*A*⋯N8^ii^	0.96	2.51	3.427 (2)	159

Symmetry codes: (i) 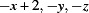; (ii) 
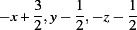
.

**Fig. 1.**
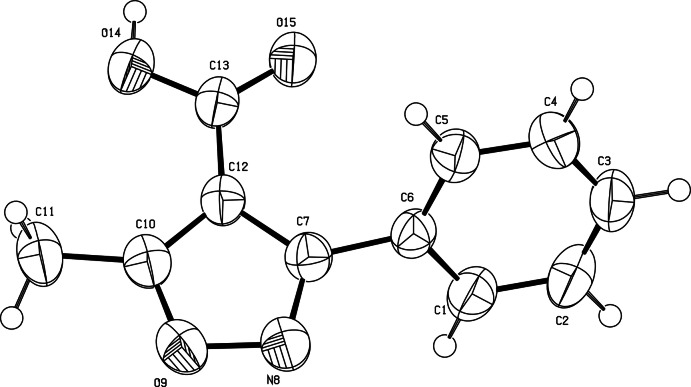
Perspective diagram of the molecule with 50% probability displacement ellipsoids.

**Fig. 2.**
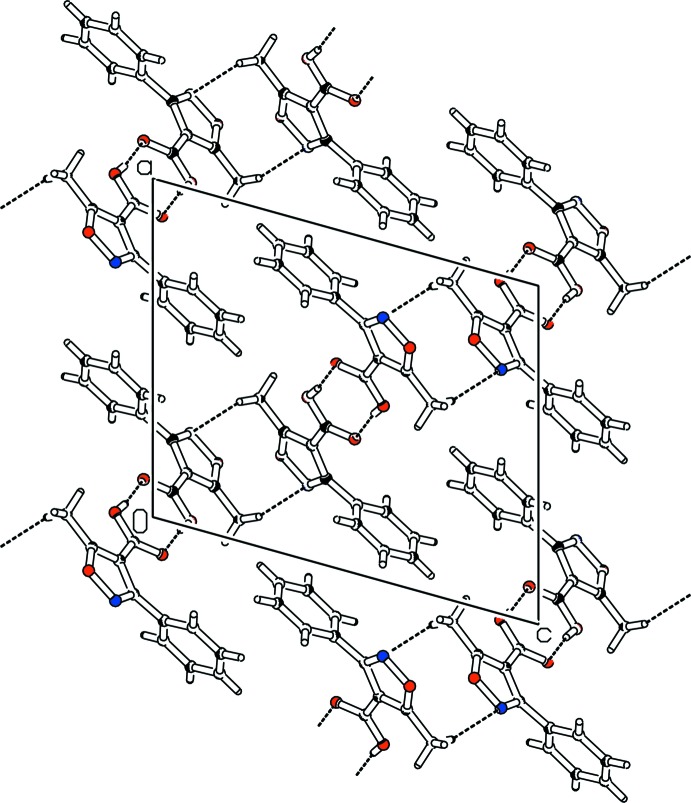
Packing diagram of the molecule viewed down the 'b' axis, showing the H-bonded dimers.

**Fig. 3.**
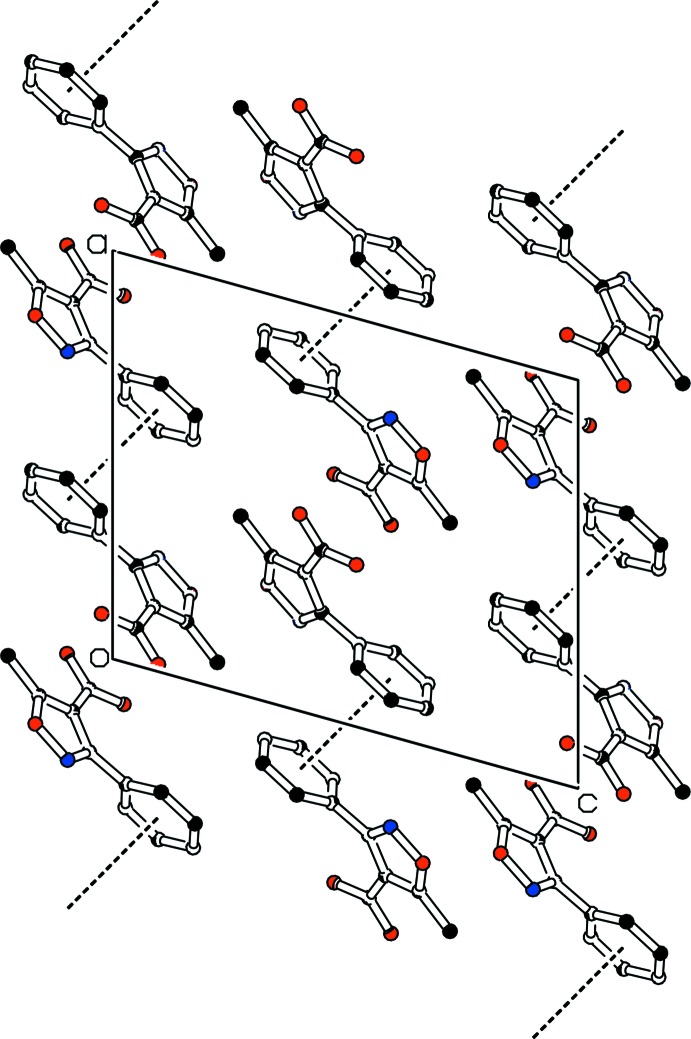
Packing diagram of the molecule viewed down the 'b' axis, showing the π–π interactions.

## supplementary crystallographic information

### Comment

Isoxazole derivatives bearing various substituents are known to have diverse biological and pharmaceutical activities; such as anti-tumor (Kang *et al.*, 2000), antiviral (Lee *et al.*, 2009), hypoglycemic (Conti *et al.*, 1998), antifungal (Basappa *et al.*, 2003) and anti-HIV activities (Shin *et al.*, 2005). In addition, isoxazoles and related compounds have attracted much interest because of their fungicidal, plant-growth regulating and antibacterial activities (Stevens & Albizati, 1984). As part of our interest in these compounds and our extensive background on isoxazole derivatives, we have synthesized the title compound to study its crystal structure.

Fig. 1 presents an ellipsoid plot of the title compound (I). The (C7/N8/O9/C10/C12) isoxazole ring is in Syn-Clinal conformation with respect to the (C1-C2-C3-C4-C5-C6) phenyl ring, as indicated by the (C1-C6-C7-N8) torsion angle of -54.40 (19)°. The carboxylic acid group at C12 is almost in the same plane as the isoxazole ring (C7-C12-C13-O15 torsion angle = -3.3 (2)°). The bond lengths and angles are within normal ranges and are comparable to related structure (Wolf *et al.*, 1995 & Chandra *et al.*, 2013). The crystal structure is stabilized by O—H···O bonds (Table 1), which define head to head dimers, and weaker C-H···N bonds (Table 1), thus defining planes parallel to (101) (Fig 2). Finally, there are π···π stacking interacions between phenyl rings with Cg···Cg[1-x,1-y,-z] and slippage displacement distances of 3.9614 (17)Å and 1.284Å respectively (Fig 3) which link planes into a 3D structure.

### Experimental

A mixture of benzaldehyde oxime (1 mmol), ethyl acetoacetate (2 mmol) and anhydrous zinc chloride (0.1 mmol) were taken in a 10 ml round bottomed flask and the contents were gradually heated to 60°C without any solvent for about one hour. After completion of the reaction (as indicated by TLC), the mixture was cooled to room temperature and ethanol was added with stirring for about 30 min. The solid ethyl 5-methyl-3-phenylisoxazole-4-carboxylate thus obtained was treated with 5% NaOH (10 ml) at room temperature for about 4hr. After completion of the reaction (as indicated by TLC), the reaction mixture was acidified with 2 N HCl. The solids thus obtained were filtered and recrystalized from hot ethanol to get crystals of the title compound.

### Refinement

H atoms were placed at idealized positions and allowed to ride on their parent atoms with C–H distances in the range of 0.93 to 0.96 Å; *U*_iso_(H) = 1.2*U*_eq_(carrier atom) for all H atoms.

### Crystal data

**Table:**

C_11_H_9_NO_3_	*F*(000) = 424
*M**_r_* = 203.19	*D*_x_ = 1.386 Mg m^−^^3^
Monoclinic, *P*2_1_/*n*	Mo *K*α radiation, λ = 0.71073 Å
Hall symbol: -P 2yn	Cell parameters from 1712 reflections
*a* = 11.953 (4) Å	θ = 2.0–25.0°
*b* = 5.981 (2) Å	µ = 0.10 mm^−^^1^
*c* = 14.142 (5) Å	*T* = 273 K
β = 105.548 (6)°	Block, yellow
*V* = 974.0 (6) Å^3^	0.30 × 0.25 × 0.20 mm
*Z* = 4	

### Data collection

**Table:**

Bruker APEXII CCD area-detector diffractometer	*R*_int_ = 0.026
ω and φ scans	θ_max_ = 25.0°, θ_min_ = 2.0°
8619 measured reflections	*h* = −14→14
1712 independent reflections	*k* = −7→7
1558 reflections with *I* > 2σ(*I*)	*l* = −16→16

### Refinement

**Table:**

Refinement on *F*^2^	Secondary atom site location: difference Fourier map
Least-squares matrix: full	Hydrogen site location: inferred from neighbouring sites
*R*[*F*^2^ > 2σ(*F*^2^)] = 0.039	H-atom parameters constrained
*wR*(*F*^2^) = 0.111	*w* = 1/[σ^2^(*F*_o_^2^) + (0.0647*P*)^2^ + 0.1799*P*] where *P* = (*F*_o_^2^ + 2*F*_c_^2^)/3
*S* = 1.05	(Δ/σ)_max_ = 0.001
1712 reflections	Δρ_max_ = 0.19 e Å^−^^3^
138 parameters	Δρ_min_ = −0.14 e Å^−^^3^
0 restraints	Extinction correction: *SHELXL97* (Sheldrick, 2008), FC^*^=KFC[1+0.001XFC^2^Λ^3^/SIN(2Θ)]^-1/4^
Primary atom site location: structure-invariant direct methods	Extinction coefficient: 0.078 (6)

### Special details

**Table:**

Geometry. Bond distances, angles *etc*. have been calculated using the rounded fractional coordinates. All su's are estimated from the variances of the (full) variance-covariance matrix. The cell e.s.d.'s are taken into account in the estimation of distances, angles and torsion angles
Refinement. Refinement on *F*^2^ for ALL reflections except those flagged by the user for potential systematic errors. Weighted *R*-factors *wR* and all goodnesses of fit *S* are based on *F*^2^, conventional *R*-factors *R* are based on *F*, with *F* set to zero for negative *F*^2^. The observed criterion of *F*^2^ > σ(*F*^2^) is used only for calculating -*R*-factor-obs *etc*. and is not relevant to the choice of reflections for refinement. *R*-factors based on *F*^2^ are statistically about twice as large as those based on *F*, and *R*-factors based on ALL data will be even larger.

### Fractional atomic coordinates and isotropic or equivalent isotropic displacement parameters (Å^2^)

**Table:**

	*x*	*y*	*z*	*U*_iso_*/*U*_eq_	
O9	0.78858 (10)	0.77608 (19)	−0.16618 (8)	0.0644 (4)	
O14	0.98209 (9)	0.19333 (19)	−0.09796 (8)	0.0626 (4)	
O15	0.89662 (9)	0.16156 (18)	0.02382 (7)	0.0583 (4)	
N8	0.72219 (12)	0.7544 (2)	−0.09692 (10)	0.0627 (5)	
C1	0.70696 (13)	0.6540 (3)	0.10434 (12)	0.0577 (5)	
C2	0.65298 (14)	0.6027 (3)	0.17680 (13)	0.0667 (6)	
C3	0.59319 (14)	0.4050 (3)	0.17315 (12)	0.0646 (6)	
C4	0.58949 (14)	0.2546 (3)	0.09855 (13)	0.0634 (5)	
C5	0.64525 (13)	0.3017 (3)	0.02718 (11)	0.0556 (5)	
C6	0.70323 (11)	0.5035 (2)	0.02904 (9)	0.0458 (4)	
C7	0.75633 (11)	0.5675 (2)	−0.05041 (10)	0.0465 (4)	
C10	0.85935 (12)	0.6006 (2)	−0.15866 (10)	0.0503 (4)	
C11	0.93670 (14)	0.6030 (3)	−0.22477 (11)	0.0643 (6)	
C12	0.84386 (10)	0.4605 (2)	−0.08704 (9)	0.0445 (4)	
C13	0.90974 (11)	0.2583 (2)	−0.05073 (10)	0.0454 (4)	
H1	0.74570	0.78940	0.10620	0.0690*	
H2	0.65710	0.70230	0.22810	0.0800*	
H3	0.55530	0.37280	0.22100	0.0780*	
H4	0.54920	0.12080	0.09630	0.0760*	
H5	0.64400	0.19820	−0.02220	0.0670*	
H11A	0.91180	0.49090	−0.27460	0.0960*	
H11B	1.01490	0.57240	−0.18750	0.0960*	
H11C	0.93350	0.74740	−0.25510	0.0960*	
H14	1.01490	0.07930	−0.07260	0.0940*	

### Atomic displacement parameters (Å^2^)

**Table:**

	*U*^11^	*U*^22^	*U*^33^	*U*^12^	*U*^13^	*U*^23^
O9	0.0729 (7)	0.0625 (7)	0.0640 (7)	0.0040 (5)	0.0289 (6)	0.0164 (5)
O14	0.0661 (7)	0.0664 (7)	0.0657 (7)	0.0140 (5)	0.0357 (5)	0.0054 (5)
O15	0.0618 (6)	0.0630 (7)	0.0569 (6)	0.0092 (5)	0.0275 (5)	0.0080 (5)
N8	0.0654 (8)	0.0622 (8)	0.0683 (8)	0.0096 (6)	0.0312 (7)	0.0138 (6)
C1	0.0544 (8)	0.0569 (9)	0.0676 (9)	−0.0019 (7)	0.0264 (7)	−0.0099 (7)
C2	0.0636 (9)	0.0795 (11)	0.0660 (10)	0.0025 (8)	0.0328 (8)	−0.0163 (8)
C3	0.0604 (9)	0.0801 (11)	0.0630 (9)	0.0064 (8)	0.0332 (7)	0.0068 (8)
C4	0.0634 (9)	0.0615 (9)	0.0722 (10)	−0.0058 (7)	0.0304 (8)	0.0056 (8)
C5	0.0597 (9)	0.0553 (9)	0.0559 (8)	−0.0029 (7)	0.0226 (7)	−0.0044 (7)
C6	0.0397 (7)	0.0511 (8)	0.0488 (7)	0.0059 (5)	0.0155 (5)	0.0022 (6)
C7	0.0442 (7)	0.0481 (7)	0.0483 (7)	−0.0006 (6)	0.0142 (6)	0.0001 (6)
C10	0.0513 (7)	0.0547 (8)	0.0459 (7)	−0.0069 (6)	0.0147 (6)	−0.0019 (6)
C11	0.0708 (10)	0.0761 (11)	0.0535 (9)	−0.0162 (8)	0.0297 (8)	−0.0023 (7)
C12	0.0430 (7)	0.0496 (7)	0.0425 (7)	−0.0052 (5)	0.0145 (5)	−0.0040 (5)
C13	0.0432 (7)	0.0510 (8)	0.0449 (7)	−0.0037 (5)	0.0169 (5)	−0.0059 (6)

### Geometric parameters (Å, º)

**Table:**

O9—N8	1.4222 (19)	C7—C12	1.4363 (19)
O9—C10	1.3342 (18)	C10—C12	1.3649 (19)
O14—C13	1.2863 (18)	C10—C11	1.481 (2)
O15—C13	1.2488 (17)	C12—C13	1.4593 (18)
O14—H14	0.8200	C1—H1	0.9300
N8—C7	1.3057 (18)	C2—H2	0.9300
C1—C2	1.384 (2)	C3—H3	0.9300
C1—C6	1.386 (2)	C4—H4	0.9300
C2—C3	1.375 (3)	C5—H5	0.9300
C3—C4	1.378 (3)	C11—H11A	0.9600
C4—C5	1.380 (2)	C11—H11B	0.9600
C5—C6	1.389 (2)	C11—H11C	0.9600
C6—C7	1.4813 (19)		
			
O9···C10^i^	3.268 (2)	C10···O15^iii^	3.345 (2)
O9···C11^i^	3.351 (2)	C10···C13^iii^	3.566 (2)
O14···O15^ii^	2.6252 (18)	C11···O14	2.999 (2)
O14···C11	2.999 (2)	C11···O9^iv^	3.351 (2)
O15···C11^iii^	3.316 (2)	C11···N8^iv^	3.427 (2)
O15···O14^ii^	2.6252 (18)	C11···O15^iii^	3.316 (2)
O15···C13^ii^	3.367 (2)	C12···C13^iii^	3.496 (2)
O15···C5	3.132 (2)	C13···O15^ii^	3.367 (2)
O15···C6	3.102 (2)	C13···C10^iii^	3.566 (2)
O15···C10^iii^	3.345 (2)	C13···C12^iii^	3.496 (2)
O9···H11A^i^	2.6500	C13···H14^ii^	2.6600
O14···H11B	2.6800	H1···N8	2.8200
O14···H14^ii^	2.9000	H11A···O9^iv^	2.6500
O15···H11B^iii^	2.7700	H11A···N8^iv^	2.5100
O15···H14^ii^	1.8100	H11B···O14	2.6800
N8···C11^i^	3.427 (2)	H11B···O15^iii^	2.7700
N8···H1	2.8200	H14···O14^ii^	2.9000
N8···H11A^i^	2.5100	H14···O15^ii^	1.8100
C5···O15	3.132 (2)	H14···C13^ii^	2.6600
C6···O15	3.102 (2)	H14···H14^ii^	2.3700
C10···O9^iv^	3.268 (2)		
			
N8—O9—C10	109.41 (11)	O14—C13—C12	116.24 (12)
C13—O14—H14	109.00	O15—C13—C12	120.20 (12)
O9—N8—C7	105.56 (12)	O14—C13—O15	123.54 (12)
C2—C1—C6	119.97 (16)	C2—C1—H1	120.00
C1—C2—C3	120.23 (16)	C6—C1—H1	120.00
C2—C3—C4	119.98 (16)	C1—C2—H2	120.00
C3—C4—C5	120.28 (16)	C3—C2—H2	120.00
C4—C5—C6	120.02 (15)	C2—C3—H3	120.00
C1—C6—C7	118.88 (12)	C4—C3—H3	120.00
C5—C6—C7	121.56 (12)	C3—C4—H4	120.00
C1—C6—C5	119.49 (13)	C5—C4—H4	120.00
N8—C7—C6	117.63 (12)	C4—C5—H5	120.00
N8—C7—C12	111.06 (12)	C6—C5—H5	120.00
C6—C7—C12	131.31 (11)	C10—C11—H11A	109.00
O9—C10—C11	115.59 (12)	C10—C11—H11B	109.00
O9—C10—C12	109.44 (12)	C10—C11—H11C	110.00
C11—C10—C12	134.94 (13)	H11A—C11—H11B	110.00
C7—C12—C13	128.23 (11)	H11A—C11—H11C	109.00
C10—C12—C13	127.03 (12)	H11B—C11—H11C	109.00
C7—C12—C10	104.53 (11)		
			
C10—O9—N8—C7	0.33 (15)	C1—C6—C7—C12	124.36 (16)
N8—O9—C10—C11	−178.39 (12)	C5—C6—C7—N8	122.44 (15)
N8—O9—C10—C12	−0.04 (15)	N8—C7—C12—C10	0.45 (16)
O9—N8—C7—C12	−0.47 (15)	N8—C7—C12—C13	175.43 (13)
O9—N8—C7—C6	178.53 (11)	C6—C7—C12—C10	−178.37 (14)
C2—C1—C6—C5	−0.1 (2)	C6—C7—C12—C13	−3.4 (2)
C6—C1—C2—C3	−1.6 (3)	O9—C10—C12—C7	−0.23 (15)
C2—C1—C6—C7	176.80 (14)	O9—C10—C12—C13	−175.28 (12)
C1—C2—C3—C4	1.7 (3)	C11—C10—C12—C7	177.67 (16)
C2—C3—C4—C5	−0.2 (3)	C11—C10—C12—C13	2.6 (3)
C3—C4—C5—C6	−1.5 (3)	C7—C12—C13—O14	178.55 (13)
C4—C5—C6—C7	−175.22 (14)	C7—C12—C13—O15	−3.3 (2)
C4—C5—C6—C1	1.6 (2)	C10—C12—C13—O14	−7.5 (2)
C1—C6—C7—N8	−54.40 (19)	C10—C12—C13—O15	170.61 (13)
C5—C6—C7—C12	−58.8 (2)		

Symmetry codes: (i) −*x*+3/2, *y*+1/2, −*z*−1/2; (ii) −*x*+2, −*y*, −*z*; (iii) −*x*+2, −*y*+1, −*z*; (iv) −*x*+3/2, *y*−1/2, −*z*−1/2.

### Hydrogen-bond geometry (Å, º)

**Table:**

*D*—H···*A*	*D*—H	H···*A*	*D*···*A*	*D*—H···*A*
O14—H14···O15^ii^	0.82	1.81	2.6252 (18)	172
C11—H11*A*···N8^iv^	0.96	2.51	3.427 (2)	159

Symmetry codes: (ii) −*x*+2, −*y*, −*z*; (iv) −*x*+3/2, *y*−1/2, −*z*−1/2.
